# Mammographic density and risk of breast cancer by mode of detection and tumor size: a case-control study

**DOI:** 10.1186/s13058-016-0722-4

**Published:** 2016-06-18

**Authors:** Kavitha Krishnan, Laura Baglietto, Carmel Apicella, Jennifer Stone, Melissa C. Southey, Dallas R. English, Graham G. Giles, John L. Hopper

**Affiliations:** Centre for Epidemiology and Biostatistics, Melbourne School of Population and Global Health, University of Melbourne, Level 3, 207 Bouverie Street, Carlton, VIC 3053 Australia; Cancer Epidemiology Centre, Cancer Council Victoria, Melbourne, Australia; Centre for Genetic Origins of Health and Disease, Curtin University and University of Western Australia, Crawley, Australia; Department of Epidemiology and Preventive Medicine, Monash University, Melbourne, Australia; Université Paris-Saclay, Univ. Paris-Sud, UVSQ, CESP, INSERM, Villejuif, France; Gustave Roussy, F-94805 Villejuif, France; Genetic Epidemiology Laboratory, Department of Pathology, University of Melbourne, Melbourne, Australia; Seoul Department of Epidemiology, School of Public Health, Seoul National University, Seoul, Korea; Institute of Health and Environment, Seoul National University, Seoul, Korea

**Keywords:** Mammographic density, Breast cancer, Detection mode, Tumor size

## Abstract

**Background:**

Risk of screen-detected breast cancer mostly reflects inherent risk, while risk of interval cancer reflects inherent risk and risk of masking (risk of the tumor not being detected due to increased dense tissue). Therefore the predictors of whether a breast cancer is interval or screen-detected include those that predict masking. Our aim was to investigate the associations between mammographic measures and (1) inherent risk, and (2) masking.

**Methods:**

We conducted a case-control study nested within the Melbourne collaborative cohort study of 244 screen-detected cases (192 small tumors (<2 cm)) matched to 700 controls and 148 interval cases (76 small tumors) matched to 446 controls. Dense area (DA), percent dense area (PDA), and non-dense area (NDA) were measured using the Cumulus software. Conditional and unconditional logistic regression were applied as appropriate to estimate the odds per adjusted standard deviation (OPERA) adjusted for age and body mass index (BMI), allowing for the association with BMI to be a function of age at diagnosis. Tests of fit were performed using the Bayesian information criterion (BIC) and the area under the receiver operating characteristic curve.

**Results:**

For screen-detected cancer, the association with BMI had a marginally significant dependence on age at diagnosis, and after adjustment both DA and PDA were associated with risk (OPERA approximately 1.2) and gave a similar fit. NDA was not associated with risk. For interval cancer, the BMI risk association was not dependent on age at diagnosis and the best fitting model was PDA alone (OPERA = 2.24, 95 % confidence interval 1.75, 2.86). Prediction of interval versus screen-detected cancer was best achieved by PDA alone (OPERA = 1.76, 95 % confidence interval 1.39, 2.22) with no association with BMI. When the analysis was restricted to small tumors to reduce the influence of tumor growth, we obtained similar results.

**Conclusions:**

Inherent breast cancer risk is predicted by BMI and DA or PDA, but not NDA. Masking is predicted by PDA, and not by BMI. Understanding risk and masking could help tailor mammographic screening.

**Electronic supplementary material:**

The online version of this article (doi:10.1186/s13058-016-0722-4) contains supplementary material, which is available to authorized users.

## Background

The regions of the breast that appear white or bright on a mammogram are referred to as being mammographically dense, and are usually measured in terms of their absolute area on the mammogram (dense area, DA), or in terms of the percentage of the total area on the mammogram covered by dense area (percent dense area, PDA). These mammographic density (MD) measures, after adjustment for age and body mass index (BMI) due to negative confounding, are positively associated with risk of developing breast cancer [1–5]. There are also suggestions of a positive association between MD and risk of masking of breast tumors [1, 2, 5] and rate of tumor growth [6]. Masking of a breast tumor is defined as a tumor being hidden on a mammogram and not being detected due to the similar appearances of both mammographically dense regions and the tumor, thus, decreasing the sensitivity of mammography.

The mammographic regions that appear non-white are referred to as non-dense area (NDA), and their negative association with overall risk of breast cancer - without differentiation between risk of developing breast cancer, masking and growth rate - is controversial [3, 7]. NDA is presumed to represent mostly fat tissue; however, even after adjusting for BMI and DA, with which it is negatively correlated, it has been found to be negatively associated with breast cancer risk [7]. The interpretation of this finding is not obvious, especially given the well-documented involvement of adiposity in the postmenopausal period in pathways triggering aromatase expression and increasing postmenopausal risk [8]. Also, DA and NDA are typically negatively correlated, so the aforementioned associations in different directions could just be reflecting “both sides of the same coin”, as we postulated in our previous paper [7]. 

Therefore, the concurrent associations between developing and masking of a breast tumor and the different mammography measures, DA, PDA and NDA, is unclear. The role of different rates of tumor growth also poses additional challenges.

The risk of a woman being diagnosed with breast cancer, given age, BMI and MD measures, is the combination of: (1) her inherent risk of developing breast cancer; (2) her risk of having any existing tumor masked; and (3) the growth rate of her tumor should she develop one. We assumed the risk of screen-detected breast cancer is mostly influenced by inherent risk, while risk of interval breast cancer is due to a combination of inherent risk and risk of masking. Therefore, given a woman is diagnosed with breast cancer, the factors that differentiate her having a screen-detected versus an interval cancer will mostly be those that influence risk of masking. Restricting the analysis to small tumors should lessen the influence on the latter due to the growth rate of tumors.

We have previously reported on the associations between DA, PDA and NDA, and breast cancer risk, while allowing for the associations with BMI to vary with age at diagnosis, using a case-control study nested within the Melbourne collaborative cohort study (MCCS) [7]. Here we have used the same study to investigate the risks of developing breast tumors and the risk of masking, by analyzing cases by tumor detection mode and tumor size. Tumor detection mode was categorized as screen-detected (defined as being detected at a scheduled screening) or interval (defined as being detected after a negative screening and before the next scheduled screening). We estimated inherent risk by comparing screen-detected cases with their matched controls, and risk of masking by comparing interval cases with screen-detected cases. In order to minimize the effect of tumor growth, we conducted analyses stratified by tumor size.

## Methods

The MCCS is a prospective cohort study of 41,514 people (24,469 women) aged between 27 and 76 years at study entry (99.3 % of whom were aged 40–69 years). Participants were recruited between 1990 and 1994 from the Melbourne metropolitan area. In 2009, through a record linkage between the MCCS and BreastScreen Victoria, a population-based screening program, we identified 20,444 (84 %) women in the MCCS who had attended BreastScreen Victoria at least once and were eligible for this study.

We then designed a nested case-control study using incidence density sampling. Cases were women with a first diagnosis of invasive adenocarcinoma of the breast (International Classification of Diseases for Oncology codes C50.0–C50.9). Each case was matched randomly to four controls by year of birth, year of entry into the MCCS and country of origin (Australia/New Zealand/United Kingdom/others, Italy, or Greece). We selected the mammogram closest to baseline and of the contralateral breast with respect to the laterality of the tumor in the matching case. Only craniocaudal-projection images were used in this study. Further details about the nested case-control study based on the MCCS have been published elsewhere [7, 9].

Screen-detected cases were identified at BreastScreen Victoria and interval cases were defined as those diagnosed within 2 years of a negative screening at BreastScreen Victoria (the recommendation for mammographic screening for breast cancer in Australia is biennial). The cases were further categorized by tumor size as small tumors (<2 cm) and large tumors (≥2 cm), given that breast cancer stage is based on cutoffs of 2 cm or 5 cm. For this study, we excluded 61 screen-detected cases detected at their first screening and 52 cases diagnosed more than 2 years after a negative screen.

There were 244 screen-detected cases (192 (79 %) with small tumors; 49 (20 %) with large tumors) matched to 700 controls and 148 interval cases (76 (51 %) with small tumors; 65 (44 %) with large tumors) matched to 446 controls.

### Statistical analyses

We estimated associations between the mammographic measures and risk according to the following different models: (1) BMI only; (2) BMI and a function of DA and NDA, with either as a linear combination or PDA; (3) BMI and only DA; and (4) all of the above models with mammographic measures without including BMI. The association between BMI and risk was fitted as a function of age at diagnosis of the case as a reference age, see below. BMI was measured at baseline attendance.

To compare the strength of risk factors, in the sense of how well they discriminate cases from controls, we presented model estimates in terms of odds per adjusted standard deviation (OPERA) [10], which is the risk associated with increase in the risk factor *X* (holding all other factors taken into account either in the design or model) on the scale of 1 (standard deviation) SD of *X* after adjusting the mean of *X* for all the other variables taken into account either by design or adjustment. This allows statistically independent comparisons of the disease-discrimination power of each of the different risk factors, as recently demonstrated [11, 12].

The Box-Cox method was applied in the controls to identify the appropriate transformations of the mammographic measures to achieve approximate normal distributions; DA and PDA were transformed to (DA^0.2^-1)/0.2 and (PDA^0.2^-1)/0.2, respectively, while NDA was transformed to (NDA^0.5^-1)/0.5. Each transformed mammographic measure was adjusted for age at mammogram, BMI (standardized according to the controls) and all the matching variables by fitting linear regression, and the standardized residuals were obtained.

To estimate the OPERA associated with each mammographic measure, conditional logistic regression was fitted adjusting for age at mammogram with the standardized residuals corresponding to each mammographic measure, separately, for screen-detected and interval cancers. Letting *r* be the correlation between the standardized residuals of DA and NDA (denoted as DA’ and NDA’, respectively), when fitting together DA’ and NDA’ in the model, to obtain the OPERA of DA’ we multiplied log(OPERA) of DA’ with [(1-r^2^)]^0.5^, which is the standard deviation of DA’ after adjusting for NDA’. Similarly, we obtained the OPERA for NDA’.

BMI measured at cohort entry was standardized according to the mean and SD of the controls. To allow the association between BMI and risk to be dependent on age, an interaction term between the standardized BMI and reference age (age at diagnosis for the case and for its matched controls) was fitted in the models. The likelihood ratio test was applied to test the significance of the interaction between BMI and reference age.

To estimate the OPERA for having interval versus screen-detected breast cancer we fitted unconditional logistic regression to data from cases only. BMI and all three mammographic measures were included in the same format as mentioned above and the models were adjusted for age at mammogram. For these analyses we presented only the estimates when fitting BMI as a constant because we found no evidence that the association between BMI and mode of detection depended on age at diagnosis.

Relative goodness of fit was assessed by the Bayesian information criterion (BIC), and by the area under the receiver operating characteristic curve (AUC). We also tested for differences between AUCs using De Long’s test [13]. To compare the estimates corresponding to risk of small tumors and large tumors we applied the Student’s two-sided *t* test, assuming independence of normally distributed log(OPERA) estimates with a standard deviation consistent with the width of the confidence interval (CI). There is a slight overlap in the datasets used to estimate risk of small and large tumors due to the design properties of the nested case-control study and therefore, there is a possibility of overestimation of a significant difference.

We conducted sensitivity analyses using unconditional logistic regression in which we made further adjustments for the following potential confounders that were assessed at cohort entry: BMI at age 18–21 years; age at menarche; parity and lactation; menopausal status; use of hormone replacement therapy (HRT); use of oral contraceptives (OC); alcohol consumption and energy intake; and the matching variables (country of birth, year of birth, year of cohort entry and reference age). These analyses were also repeated using only those women who had undergone mammography within 5 years of cohort entry.

We also conducted the following sensitivity analyses: (1) excluding cases diagnosed between 1 and 2 years after negative screening, and their matched controls; (2) excluding ever-users of HRT; and (3) excluding cases diagnosed within 2 years of the mammogram, and their matching controls. Statistical analyses were performed using Stata 12.1 (Stata Corporation, College Station, TX). A two-sided *P* value <0.05 was considered to be nominally statistically significant.

## Results

Table 1 presents characteristics of the study sample, and shows that there were no differences between cases and controls in age at which mammography was performed, either by detection mode or by detection mode and tumor size. Screen-detected cases were on average older than interval cases when diagnosed (65 years vs 62 years, *P* < 0.001), older at baseline when covariates were measured (56 years vs 54 years, *P* = 0.01), and older when the mammogram closest to study entry was performed (59 years vs 57 years, *P* < 0.01).

**Table 1:** Characteristics of study participants

	Screen-detected cases			Interval cases			
	Cases (n = 244)	Controls (n = 700)	*P*	Cases (n = 148)	Controls (n = 446)	*P*	*P* ^a^
Age at baseline, years	56 (8)	56 (8)	0.72	54 (8)	54 (8)	0.72	0.01
Age at mammogram, years	59 (7)	59 (7)	0.82	57 (7)	57 (7)	0.79	<0.01
Age at diagnosis, years	65 (7)			62 (7)			<0.001
Time between age at mammogram and reference age, years	6 (3)	6 (3)	0.58	5 (3)	5 (4)	0.93	0.12
Total energy intake, MJ/day	8.4 (2.9)	8.6 (3.5)	0.40	8.7 (3.2)	8.7 (3.1)	0.91	0.30
BMI, kg/m^2^							
All women	27.5 (4.9)	26.7 (4.9)	0.04	26.7 (5.3)	26.5 (4.8)	0.68	0.11
Premenopausal	27.2 (5.7)	26.2 (5.0)	0.17	25.9 (4.9)	26.1 (4.6)	0.72	0.15
Postmenopausal	27.6 (4.6)	27.0 (4.9)	0.10	27.2 (5.5)	26.7 (5.0)	0.41	0.51
BMI at age 18–21 years, kg/m^2^	21.5 (2.9)	21.5 (2.9)	0.86	21.4 (2.5)	21.5 (2.8)	0.54	0.66
Breast							
Total area, cm^2^	143.7 (60.9)	137.8 (57.6)	0.17	125.0 (56.9)	133.4 (60.3)	0.14	<0.01
Non-dense area, cm^2^	124.2 (62.1)	120.9 (58.8)	0.47	96.5 (55.6)	115.3 (60.6)	<0.001	<0.0001
Dense area, cm^2^	19.6 (21.7)	16.8 (20.6)	0.08	28.5 (24.5)	18.1 (19.4)	<0.0001	<0.001
Percent dense area	15.5 (15.7)	14.0 (15.3)	0.18	25.2 (17.9)	15.4 (14.7)	<0.0001	<0.0001
Country of birth, *n* (%)							
Anglo Saxon/other	204 (84)	583 (83)	0.99	123 (83)	371 (83)	0.99	0.70
Italy	25 (10)	74 (11)		13 (9)	40 (9)		
Greece	15 (6)	43 (6)		12 (8)	35 (8)		
Age at menarche, years *n* (%)							
<12	47 (19)	125 (18)	0.92	31 (21)	75 (17)	0.69	0.74
12	52 (21)	145 (21)		25 (17)	86 (19)		
13	55 (23)	169 (24)		36 (24)	111 (25)		
14+	88 (36)	261 (37)		56 (38)	174 (39)		
Parity and lactation, *n* (%),							
Nulliparous	38 (16)	84 (12)	0.03	25 (17)	62 (14)	0.63	0.22
Parous, never lactated	10 (4)	61 (9)		12 (8)	34 (8)		
Parous, lactated	190 (78)	542 (77)		108 (73)	343 (77)		
Menopausal status *n* (%)							
Premenopausal	69 (28)	195 (28)	0.88	59 (40)	170 (38)	0.72	0.02
Postmenopausal	174 (71)	504 (72)		89 (60)	275 (62)		
Hormone replacement therapy use, *n* (%)							
Never	169 (69)	493 (70)	0.75	98 (66)	323 (72)	0.17	0.55
Ever	74 (30)	205 (29)		49 (33)	122 (27)		
Oral contraceptive use, *n* (%)							
Never	93 (38)	283 (40)	0.53	58 (39)	157 (35)	0.4	0.86
Ever	150 (61)	415 (59)		90 (61)	287 (64)		
Alcohol consumption, *n* (%)							
Lifetime abstainers	109 (45)	257 (37)	0.10	43 (29)	160 (36)	0.3	<0.01
Ex-drinkers	13 (5)	24 (3)		3 (2)	13 (3)		
Low intake, 1–19 g/day	97 (40)	337 (48)		78 (53)	223 (50)		
Medium intake, 20–39 g/day	19 (8)	61 (9)		20 (14)	38 (9)		
High intake, ≥40 g/day	6 (2)	21 (3)		4 (3)	12 (3)		
Family history of breast cancer^b^, *n* (%)							
No	185 (76)	548 (78)	0.08	101 (68)	341 (76)	<0.001	0.18
Yes	38 (16)	77 (11)		30 (20)	43 (10)		
ER, *n* (%)							
Positive	188 (77.0)			94 (63.5)			<0.01
Negative	45 (18.4)			45 (30.4)			
PR, *n* (%)							
Positive	126 (51.6)			57 (38.5)			0.02
Negative	106 (43.4)			80 (54.1)			
HER2, *n* (%)							
Positive	73 (29.9)			41 (27.7)			0.64
Negative	153 (62.7)			96 (64.9)			
Grade *n* (%)							
Well-differentiated	62 (25.4)			24 (16.2)			<0.01
Moderately differentiated	104 (42.6)			52 (35.1)			
Poorly differentiated	65 (26.6)			61 (41.2)			
Nodal status, *n* (%)							
Positive	39 (16.0)			65 (43.9)			<0.001
Negative	190 (77.9)			71 (48.0)			
Tumor size, *n* (%)							
<1 cm	76 (31.1)			25 (16.9)			<0.001
1–2 cm	116 (47.5)			51 (34.5)			
≥2 cm	49 (20.1)			65 (43.9)			

Results are presented as mean (SD) unless stated otherwise. *P* values are for Pearson’s *χ*2 test or Fisher’s exact test for categorical variables and Student’s two-sided *t* test for continuous variables. ^a^ *P* values are for tests comparing screen-detected cases with interval cases. ^b^Family history of breast cancer is defined as having any relative with breast cancer. *BMI* body mass index, *SD* standard deviation, *ER* estrogen receptor, *PR* progesterone receptor, HER2 human epidermal growth factor receptor-2

Screen-detected cases had on average similar DA and PDA compared to controls (*P* = 0.08 and 0.18, respectively). Interval cases had on average greater DA and PDA and lesser NDA compared to controls (*P* < 0.001). Compared to screen-detected cases, interval cases had on average greater DA and PDA and lesser total breast area and NDA (*P* < 0.01). Among screen-detected cases, those with small tumors had on average lesser DA (*P* = 0.01) but not lesser PDA (*P* = 0.11) than those with large tumors. Similarly, among interval cases, those with small tumors had on average lesser DA (*P* < 0.01) but not lesser PDA (*P* = 0.26) than those with large tumors. Screen-detected cases had a greater BMI than the controls (*P* = 0.04), whereas there was no significant difference in BMI between interval cases and controls (*P* = 0.68). Of the women who answered the question about history of family breast cancer, the proportion who reported any family history of breast cancer was higher among those with interval and screen-detected cancers (20 % and 16 %, respectively) than it was among their respective controls (10 % and 11 %, respectively) (*P* < 0.001 and *P* = 0.08, respectively), although the difference between screen-detected cases and controls was marginally significant. Among screen-detected cases there was a greater percentage of women who had no children compared to controls (16 % vs 12 %, *P* =0.03).

In terms of tumor characteristics, interval cases were diagnosed with more tumors with poorer prognosis than screen-detected cases; estrogen receptor (ER)-negative (ER-) (30 % vs 18 %, *P* < 0.01), progesterone receptor (PR)-negative (PR-) (54 % vs 43 %, *P* = 0.02), poorly differentiated tumors (41 % vs 27 %, *P* < 0.01), positive nodal status (44 % vs 16 %, *P* < 0.001), and larger tumor size, ≥2 cm (44 % vs 20 %, *P* < 0.001).

Table 2 shows that the association between BMI and risk of screen-detected breast cancer was almost null at 50 years and increased with age at diagnosis in all models by about 30 % from 50 to 70 years, but the interaction between BMI and age was marginally significant (0.09 ≤ *P* ≤ 0.12). Both DA and PDA were positively associated with the risk of screen-detected breast cancer with a similar increase in risk of about 20 % per adjusted SD in all models. Models including either DA or PDA gave the best fit (BIC = 647 and BIC = 646, respectively). NDA was not associated with risk of screen-detected cancer in any model.

**Table 2:** Risk of breast cancer according to BMI and mammographic measures, by detection mode

		OPERA (95 % CI)						
		BMI	BMI + DA + NDA	BMI + PDA	BMI + DA	DA + NDA	PDA	DA
Screen-detected cases	BIC, AUC	653, 0.63	660, 0.65	653, 0.65	654, 0.65	654, 0.62	646, 0.63	647, 0.62
(244 cases/700 controls)	BMI per 1 SD							
	At age 50 years	0.92 (0.66, 1.27)	0.93 (0.67, 1.30)	0.94 (0.68, 1.30)	0.94 (0.67, 1.30)			
	At age 70 years	1.29 (1.07, 1.56)	1.30 (1.07, 1.57)	1.29 (1.07, 1.56)	1.30 (1.07, 1.57)			
	*P* for interaction^a^	0.09	0.11	0.12	0.11			
	DA per adjusted 1 SD		1.19 (1.04, 1.38)		1.20 (1.04, 1.39)	1.19 (1.03, 1.37)		1.20 (1.04, 1.39)
	PDA per adjusted 1 SD			1.22 (1.05, 1.41)			1.22 (1.05, 1.41)	
	NDA per adjusted 1 SD		0.95 (0.82, 1.11)			0.95 (0.82, 1.11)		
Interval cases	BIC, AUC	420, 0.60	376, 0.76	373, 0.75	388, 0.72	364, 0.75	361, 0.75	377, 0.72
(148 cases/446 controls)	BMI per 1 SD							
	At age 50 years	0.92 (0.63, 1.35)	0.81 (0.54, 1.22)	0.80 (0.54, 1.20)	0.82 (0.54, 1.23)			
	At age 70 years	1.12 (0.84, 1.49)	1.12 (0.82, 1.52)	1.10 (0.81, 1.49)	1.09 (0.81, 1.46)			
	*P* for interaction^a^	0.49	0.29	0.29	0.33			
	DA per adjusted 1 SD		1.89 (1.51, 2.36)		1.90 (1.53, 2.37)	1.87 (1.49, 2.33)		1.88 (1.52, 2.34)
	PDA per adjusted 1 SD			2.27 (1.77, 2.92)			2.24 (1.75, 2.86)	
	NDA per adjusted 1 SD		0.63 (0.50, 0.78)			0.63 (0.51, 0.78)		

All of the estimates from conditional logistic regression were adjusted for age at mammogram and the variables included into the model. *AUC* area under the receiver operating characteristic curve, *BIC* Bayesian information criterion, *BMI* body mass index, *CI* confidence interval, *DA* dense area, *NDA* non-dense area, *OPERA* odds per adjusted standard deviation, *PDA* percent dense area, *SD* standard deviation. ^a^Likelihood ratio test for the interaction with age at diagnosis

Table 2 shows a different set of results for risk of interval breast cancer. First, there was no evidence that the association between BMI and risk depended on age at diagnosis (*P* ≥ 0.29). The best fitting model under the BIC involved PDA alone, with an increase in risk of about 124 % (95 % CI 75 %, 186 %) per adjusted SD.

Table 3 shows that the association between BMI and risk of small screen-detected breast cancers increased by 32 % from 50 to 70 years although the association was only marginally dependent on age at diagnosis (0.10 ≤ *P* ≤ 0.12). The positive association with DA and PDA remained but the risk estimates were about 10 % per adjusted SD and marginally significant. In contrast, risk of large screen-detected cancers was not associated with BMI, whether fitted as dependent or independent of age at diagnosis (results not shown). The association between risk and DA or PDA was 59 % and 66 % per adjusted SD, respectively. The differences in the association between risk and DA or PDA according to size of tumor were nominally significant. There was no association between NDA and risk of small or large screen-detected breast cancer.

**Table 3:** Risk of breast cancer according to BMI and mammographic measures, by detection mode and tumor size

		OPERA (95 % CI)						
		BMI	BMI + DA + NDA	BMI + PDA	BMI + DA	DA + NDA	PDA	DA
Screen-detected, small tumors	BIC, AUC	516, 0.64	528, 0.65	521, 0.65	521, 0.65	521, 0.62	515, 0.62	515, 0.62
(192 cases/552 controls)	BMI per 1 SD							
	At age 50 years	0.91 (0.64, 1.31)	0.92 (0.64, 1.33)	0.93 (0.65, 1.33)	0.93 (0.65, 1.33)			
	At age 70 years	1.32 (1.07, 1.62)	1.32 (1.08, 1.62)	1.32 (1.07, 1.62)	1.32 (1.07, 1.62)			
	*P* for interaction^a^	0.10	0.11	0.12	0.12			
	DA per adjusted 1 SD		1.09 (0.93, 1.28)		1.10 (0.93, 1.29)	1.08 (0.92, 1.28)		1.09 (0.93, 1.28)
	PDA per adjusted 1 SD			1.12 (0.94, 1.32)			1.11 (0.94, 1.31)	
	NDA per adjusted 1 SD		0.96 (0.82, 1.13)			0.97 (0.82, 1.14)		
Screen-detected, large tumors	BIC, AUC	145, 0.60	147, 0.69	142, 0.69	142, 0.69	136, 0.69	132, 0.69	132, 0.69
(49 cases/141 controls)	BMI per 1 SD							
	At age 50 years	0.78 (0.32, 1.92)	0.83 (0.32, 2.14)	0.83 (0.33, 2.10)	0.79 (0.30, 2.07)			
	At age 70 years	1.14 (0.71, 1.83)	1.09 (0.64, 1.84)	1.10 (0.65, 1.85)	1.11 (0.66, 1.87)			
	*P* for interaction^a^	0.49	0.65	0.63	0.56			
	DA per adjusted 1 SD		1.56 (1.12, 2.19)		1.58 (1.12, 2.22)	1.57 (1.12, 2.20)		1.59 (1.13, 2.24)
	PDA per adjusted 1 SD			1.64 (1.13, 2.38)			1.66 (1.14, 2.40)	
	NDA per adjusted 1 SD		0.85 (0.58, 1.24)			0.84 (0.57, 1.22)		
Interval cases, small tumors	BIC, AUC	217, 0.62	208, 0.74	203, 0.74	211, 0.70	199, 0.73	194, 0.73	203, 0.69
(76 cases/224 controls)	BMI per 1 SD							
	At age 50 years	0.76 (0.43, 1.32)	0.68 (0.37, 1.25)	0.68 (0.37, 1.24)	0.70 (0.38, 1.26)			
	At age 70 years	0.88 (0.54, 1.43)	0.96 (0.58, 1.57)	0.93 (0.57, 1.51)	0.89 (0.55, 1.46)			
	*P* for interaction^a^	0.73	0.46	0.49	0.58			
	DA per adjusted 1 SD		1.65 (1.22, 2.23)		1.64 (1.22, 2.21)	1.61 (1.20, 2.17)		1.60 (1.20, 2.15)
	PDA per adjusted 1 SD			1.99 (1.43, 2.77)			1.94 (1.40, 2.68)	
	NDA per adjusted 1 SD		0.65 (0.48, 0.86)			0.65 (0.49, 0.86)		
Interval cases, large tumors	BIC, AUC	196, 0.59	163, 0.82	159, 0.82	167, 0.79	152, 0.82	148, 0.82	156, 0.79
(65 cases/204 controls)	BMI per 1 SD							
	At age 50 years	1.23 (0.67, 2.23)	0.99 (0.52, 1.87)	0.95 (0.51, 1.78)	0.99 (0.52, 1.89)			
	At age 70 years	1.11 (0.73, 1.70)	0.92 (0.54, 1.57)	0.94 (0.55, 1.60)	0.94 (0.58, 1.53)			
	*P* for interaction^a^	0.83	0.89	0.98	0.91			
	DA per adjusted 1 SD		2.77 (1.86, 4.14)		2.73 (1.87, 4.00)	2.74 (1.85, 4.05)		2.70 (1.86, 3.92)
	PDA per adjusted 1 SD			3.49 (2.19, 5.56)			3.43 (2.18, 5.41)	
	NDA per adjusted 1 SD		0.58 (0.40, 0.83)			0.58 (0.40, 0.83)		

All of the estimates from conditional logistic regression were adjusted for age at mammogram and the variables included into the model. *AUC* area under the receiver operating characteristic curve, *BIC* Bayesian information criterion, *BMI* body mass index, *CI* confidence interval, *DA* dense area, *NDA* non-dense area, *OPERA* odds per adjusted standard deviation, *PDA* percent dense area, *SD* standard deviation. ^a^Likelihood ratio test for the interaction with age at diagnosis

Table 3 also shows that risk of both small and large interval breast cancers was best fit by including PDA. Similar to the screen-detected cancers, for interval cancers, the results for small tumors were similar to those for overall tumors and association between mammographic density (MD) and risk was significantly stronger for large tumors than for small tumors.

Table 4 shows that the risk of interval vs screen-detected breast cancer was independent of BMI, and was best predicted by PDA alone. Results were similar when analysis was restricted to small tumors. Furthermore, the risk gradient with PDA was greater as a predictor of large tumors than it was as a predictor of small tumors, but the difference was not significant.

**Table 4:** Risk of interval versus screen-detected cancer according to BMI and mammographic measures

		OPERA (95 % CI)						
		BMI	BMI + DA + NDA	BMI + PDA	BMI + DA	DA + NDA	PDA	DA
All	BIC, AUC	527, 0.60	511, 0.68	508, 0.67	518, 0.65	506, 0.67	504, 0.67	514, 0.64
244 SDC/148 IC	BMI per 1 SD	0.89 (0.73, 1.09)	0.88 (0.71, 1.08)	0.88 (0.72, 1.08)	0.88 (0.72, 1.08)			
	DA per adjusted 1 SD		1.46 (1.18, 1.82)		1.52 (1.22, 1.89)	1.46 (1.17, 1.81)		1.51 (1.22, 1.87)
	PDA per adjusted 1 SD			1.76 (1.40,2.23)			1.76 (1.39,2.22)	
	NDA per adjusted 1 SD		0.67 (0.54, 0.83)			0.67 (0.54, 0.84)		
Small tumors	BIC, AUC	321, 0.65	319, 0.70	314, 0.70	320, 0.67	318, 0.68	313, 0.68	321, 0.65
192 SDC/76 IC	BMI per 1 SD	0.67 (0.49, 0.91)	0.72 (0.53, 0.98)	0.72 (0.52, 0.98)	0.68 (0.50, 0.94)			
	DA per adjusted 1 SD		1.42 (1.05, 1.93)		1.46 (1.08, 1.98)	1.45 (1.08, 1.96)		1.49 (1.10, 2.01)
	PDA per adjusted 1 SD			1.73 (1.25, 2.39)			1.79 (1.30, 2.47)	
	NDA per adjusted 1 SD		0.69 (0.53, 0.91)			0.68 (0.52, 0.89)		
Large tumors	BIC, AUC	168, 0.57	168, 0.68	166, 0.66	169, 0.63	164, 0.68	161, 0.66	164, 0.63
49 SDC/65 IC	BMI per 1 SD	1.10 (0.78,1.54)	0.98 (0.68, 1.43)	1.01 (0.70, 1.45)	1.04 (0.73, 1.47)			
	DA per adjusted 1 SD		1.44 (0.96, 2.17)		1.48 (1.00, 2.21)	1.44 (0.96, 2.16)		1.49 (1.01, 2.21)
	PDA per adjusted 1 SD			1.81 (1.16, 2.83)			1.81 (1.16, 2.82)	
	NDA per adjusted 1 SD		0.57 (0.35, 0.94)			0.58 (0.35, 0.94)		

All of the estimates from unconditional logistic regression were adjusted for age at mammogram and the variables included into the model. *AUC* area under the receiver operating characteristic curve, *BIC* Bayesian information criterion, *BMI* body mass index, *CI* confidence interval, *DA* dense area, *IC* interval cases, *NDA* non-dense area, *OPERA* odds per adjusted standard deviation, *PDA* percent dense area, *SD* standard deviation, *SDC* screen-detected cases

The findings were similar when we adjusted for all the confounders and further restricted the analysis to mammograms performed within 5 years of cohort entry. No substantial differences in estimates were observed from the sensitivity analyses (Additional file 1: Tables S1 to S9).

## Discussion

We found that the best-fitting risk models differed substantially between screen-detected and interval, or interval vs screen-detected breast cancers. Given our contention that the risk of screen-detected cancers mostly reflects inherent cancer risk, and the predictors of interval vs screen-detected disease mostly reflect predictors of masking, we conclude that after adjusting for age and BMI, both DA or PDA, but not NDA, were associated with inherent risk of breast cancer. In contrast, masking was best predicted by PDA alone, and is not predicted by BMI.

We have interpreted our risk estimates for screen-detected breast cancer to be broadly representative of woman’s inherent risk of developing a detectable breast tumor, given that the cases did not have a detectable tumor on prior mammograms. This could be a reasonable assumption based on a review [14], which found that within interval cases, which consist of true interval cases, false-negative cases (tumors not identified on mammography due to reader error) and occult tumors (tumors not identified on mammography due to high density), there was a lesser percentage of the latter two cases; false-negative cases (25–40 %) and occult tumors (8–12 %).

Our finding that the association between screen-detected cancer and BMI depends marginally on age at diagnosis is consistent with the epidemiological literature that has consistently identified a different association between BMI and risk of breast cancer for premenopausal and postmenopausal disease [15]. BMI has a negative association with risk of premenopausal disease, and a positive association with risk of postmenopausal disease. We also used the MCCS to model the temporal aspects of the latter phenomenon with a similar result [16].

After adjusting for BMI as aforementioned, either DA or PDA, but not NDA, were associated with screen-detected disease. Note that after adjusting for age and BMI, DA and PDA were highly correlated (Spearman’s rank correlation = 0.87). Therefore, it is not surprising that they were associated with similar risk gradients once risk was expressed on the age-adjusted and BMI-adjusted scale using OPERA [10]. The AUCs and BICs were similar. There were similar results for DA and/or PDA in previous and larger studies analyzing screen-detected cases, but they had not adjusted for BMI [17, 18], nor had they adjusted for BMI only as a constant [19].

Our finding that NDA was not associated with screen-detected disease is important given the controversy about the potential for NDA to be implicated in breast cancer risk [20]. When analyzing risk of interval versus screen-detected cancer, the role of NDA in predicting masking, after adjusting for DA, was in a different direction for these two negatively associated measures. This suggests that we might have been correct when we considered DA and NDA to be “two sides of the same coin” when discussing these issues previously [7].

Both DA and PDA gave a similar fit when analyzing risk of screen-detected cancer and when further restricted to small tumors. Recent findings from studies of single nucleotide polymorphisms (SNPs) associated with breast cancer risk found DA to be a better fit [21]. PDA is DA divided by total breast area, and is moderately correlated with BMI (Spearman’s rank correlation = −0.44). DA, on the other hand, has a weaker correlation with BMI (Spearman’s rank correlation = −0.28). After adjusting both measures for age and BMI, PDA was highly associated with DA, but PDA had undergone two substantial statistical procedures (division and adjustment). Consequently, PDA for age and BMI has more measurement error than DA for age and BMI.

When restricted to screen-detected cases with small tumors, it is more likely that the tumors were not present on previous mammography, in which case the risk estimates would better reflect those for inherent risk of the disease. This might explain the similarity in the risk model with the overall cases. Screen-detected large tumors, however, are plausibly more likely to have been present on previous mammography and therefore the risk of these tumors might be influenced by risk of (past) masking in addition to inherent risk and increased tumor growth rate. This is perhaps reflected by the fact that the association with BMI was not age-dependent, as we found from analyses of interval cancers, and of screen-detected versus interval cancers, which we contend are more about risk of masking.

Risk of interval cancer, on the other hand, represents a combination of risk of developing the tumor and risk of masking. This is because, based on the European guideline for quality reassurance of screening programs [22], interval tumors consist of true interval tumors, occult tumors and false-negative tumors. In our results the age dependent association between BMI and risk seemed to have a positive trend but it was not significant, which could be an indication that risk of interval cancer is not based solely on risk of developing the tumor. For small tumors, if we assume that they are mainly true interval tumors, then the results would be more representative of inherent risk but the best-fitting risk model in our study was very different to that for screen-detected small tumors. This would suggest that there was a high contribution of occult tumors and false-negative tumors among our small tumors and this might explain the similarity in results to those for interval tumors overall, as it also represents a combination of actual risk and risk of masking. Risk of large tumors, on the other hand, might be a combination of all three risks; developing the tumor, masking and rapid growth.

When comparing interval with screen-detected breast cancers, if we assume that the majority of the screen-detected tumors were present only on the mammographic examination at which the tumor was identified and not on prior mammography, then the predictors are in effect referring to risk of masking. This is supported by our finding that the association between risk and BMI did not depend on age at diagnosis. Our results suggest that the percentage, rather than the absolute amount, of “whiteness” on a mammogram is a stronger risk factor for tumor masking. Other studies that have investigated PDA (including the Breast Imaging, Reporting and Data System (BIRADS)) found similar results [23–27]. Results for DA need further investigation because unlike our study, Boyd et al. [23] found that DA was associated with increased risk of interval cases compared with screen-detected cases but the association disappeared after adjusting for NDA. Studies in which interval cases detected within one year of the negative mammogram were defined as those most influenced by masking, found that greater percent density was a stronger risk factor for masking [23, 24]. Similar results were found in our study when we restricted the analysis of interval cases with small tumors to only those detected within one year of the negative mammogram. In our study, when compared with screen-detected cases, interval cases had less total breast area and more DA and PDA, and less NDA, which might indicate features of the breast that are more predictive of masking. When restricted to small tumors, and therefore possibly reducing the influence of tumor growth, the best fitting risk model was similar to that for all tumors, and thus, the risk estimates for this subgroup of disease might be more appropriate measures of risk of masking.

Risk of large tumors is hard to interpret due to the possible influence of tumor growth. MD risk gradients were significantly greater for large tumors compared with small tumors, for both detection modes. This observation could be due to the greater influence of increased tumor growth rate on large tumors. Two other Australian studies [28, 29] with larger sample sizes also found MD to be a stronger risk factor for large vs small screen-detected cancers, but the difference was not statistically tested. One of the studies [29], however, found no association between PDA and risk of screen-detected disease with small tumors. Contrary to ours, both studies [28, 29] observed greater MD risk gradients for screen-detected large tumors than interval tumors, but again this was not statistically tested. The differences, if any, might be due to the different cutoff of 1.5 cm used to categorize tumors by size, and also to not adjusting for BMI. Overall, studies estimating the risk gradients for MD without taking into account the detection mode and tumor size might produce overestimates of risk and masking by including large tumors due to the influence of rapid tumor growth.

One strength of our study is that, as BMI is known to have differential associations with breast cancer risk [15], we realistically modelled the BMI association by allowing it to vary with age at diagnosis. BMI had been calculated from measured height and weight at cohort entry. To our knowledge, this is the first study to estimate the differential risk of developing breast cancer and risk of masking by investigating the concurrent associations with all three measures, DA, NDA and PDA, and by taking into account the detection mode and tumor size.

A limitation of our study is the sample size, especially for categories defined by detection and tumor size. We were not able to retrospectively review mammograms and identify the proportion of true interval, false-negative, and occult tumors [27]. If there were fewer occult tumors in our interval cases, the OPERA estimates corresponding to MD might be attenuated. We have also assumed the growth rate to be slower for smaller tumors. In our data, the time taken for the interval tumors to be diagnosed after the last scheduled screening was similar for small and large tumors (mean (SD), 1.08 years (0.61) and 1.00 years (0.52) respectively, *P* = 0.32). If the tumors occurred at the same time, or if we were able to test this for true interval cases, this might mean that the larger tumors were on average growing at a faster rate. Misclassification of the detection mode of cases might also have occurred if screen-detected cases were wrongly classified as false-negative interval cases while true interval cases or occult tumors were wrongly classified as screen-detected cases. Other strengths and limitations of the study were discussed in our previous report [7].

## Conclusions

In conclusion, we have gained greater insight into the roles of MD in breast cancer diagnosis by analyzing cases by their detection mode and tumor size. After properly taking into account the role of BMI as a risk factor for disease, we found that both DA or PDA were predictors of inherent risk and NDA played no role. For masking, PDA alone was the best predictor, and BMI was not a risk factor for this outcome. Consequently, screening strategies could be tailored; e.g., women with greater age-adjusted and BMI-adjusted DA, who are at higher inherent risk of the disease, could be recommended prevention strategies, early screening and/or more frequent screening, taking into account other measured risk factors such as family history. Women with greater PDA, irrespective of their BMI, who are at higher risk of masking, could be recommended for additional screening by ultrasound. Therefore, from the point of view of using MD measurements to improve screening, masking and inherent risk need to be thought of as separate, though interacting, issues.

## Abbreviations

AMDRF, Australian Mammographic Density Research Facility; AUC, area under the receiver operating characteristic curve; BIC, Bayesian information criterion; BMI, body mass index; BSV, BreastScreen Victoria; CI, confidence interval; DA, dense area; DCIS, ductal carcinoma in situ; ER, estrogen receptor; HER2, human epidermal growth factor receptor-2; HRT, hormone replacement therapy; MCCS, Melbourne collaborative cohort study; MD, mammographic density; NDA, non-dense area; OPERA, odds per adjusted standard deviation; PDA, percent dense area; PR, progesterone receptor; SD, standard deviation

## Additional file

Additional file 1:
**Table S1.** Risk of breast cancer for BMI and mammographic measures by detection mode, excluding HRT users. **Table S2** Risk of breast cancer for BMI and mammographic measures by detection mode and tumor size, excluding HRT users. **Table S3** Risk of interval versus screen-detected cancer for BMI and mammographic measures, excluding HRT users. **Table S4** Risk of breast cancer for BMI and mammographic measures by detection mode, excluding cases (and matched controls) diagnosed within 2 years of mammogram. **Table S5** Risk of breast cancer for BMI and mammographic measures by detection mode and tumor size, excluding cases (and matched controls) diagnosed within 2 years from mammogram. **Table S6** Risk of interval versus screen-detected cancer for BMI and mammographic measures, excluding cases (and matched controls) diagnosed within 2 years from mammogram. **Table S7** Risk of breast cancer for BMI and mammographic measures by detection mode, excluding cases diagnosed between 1 and 2 years after negative screening, and their matching controls. **Table S8** Risk of breast cancer for BMI and mammographic measures by detection mode and tumor size, excluding cases diagnosed between 1 and 2 years after negative screening, and their matching controls. **Table S9** Risk of interval versus screen-detected cancer for BMI and mammographic measures, excluding cases diagnosed between 1 and 2 years after negative screening, and their matching controls. (DOCX 61 kb)
