# Supplementary material for: Mammographic density and risk of breast cancer by mode of detection and tumor size: a case-control study
Source: Breast Cancer Res. 2016 Jun 18;18:63. doi: 10.1186/s13058-016-0722-4 (PMC4912759; doi:10.1186/s13058-016-0722-4)
Supplement: Additional file 1: — Table S1. Risk of breast cancer for BMI and mammographic measures by detection mode, excluding HRT users. Table S2 Risk of breast cancer for BMI and mammographic measures by detection mode and tumor size, excluding HRT users. Table S3 Risk of interval versus screen-detected cancer for BMI and mammographic measures, excluding HRT users. Table S4 Risk of breast cancer for BMI and mammographic measures by detection mode, excluding cases (and matched controls) diagnosed within 2 years of mammogram. Table S5 Risk of breast cancer for BMI and mammographic measures by detection mode and tumor size, excluding cases (and matched controls) diagnosed within 2 years from mammogram. Table S6 Risk of interval versus screen-detected cancer for BMI and mammographic measures, excluding cases (and matched controls) diagnosed within 2 years from mammogram. Table S7 Risk of breast cancer for BMI and mammographic measures by detection mode, excluding cases diagnosed between 1 and 2 years after negative screening, and their matching controls. Table S8 Risk of breast cancer for BMI and mammographic measures by detection mode and tumor size, excluding cases diagnosed between 1 and 2 years after negative screening, and their matching controls. Table S9 Risk of interval versus screen-detected cancer for BMI and mammographic measures, excluding cases diagnosed between 1 and 2 years after negative screening, and their matching controls. (DOCX 61 kb) [file 13058_2016_722_MOESM1_ESM.docx]

Table S1: Risk of breast cancer for BMI and mammographic measures by detection mode, excluding HRT users

|  |  | OPERA (95% CI) | | | | | | |
| --- | --- | --- | --- | --- | --- | --- | --- | --- |
|  |  | BMI | BMI+DA+NDA | BMI+PDA | BMI+DA | DA+NDA | PDA | DA |
| Screen detected cases | BIC, AUC | 374, 0.58 | 385, 0.59 | 378, 0.59 | 379, 0.59 | 376, 0.57 | 370, 0.57 | 371, 0.57 |
| (170 cases /495 controls) | BMI per 1 SD |  |  |  |  |  |  |  |
|  | At age 50 years | 0.78 (0.52,1.17) | 0.78 (0.52,1.18) | 0.79 (0.53,1.19) | 0.79 (0.52,1.19) |  |  |  |
|  | At age 70 years | 1.27 (0.99,1.63) | 1.27 (0.98,1.63) | 1.27 (0.99,1.63) | 1.27 (0.99,1.64) |  |  |  |
|  | P for interaction^a^ | 0.06 | 0.06 | 0.06 | 0.06 |  |  |  |
|  | DA per *adjusted* 1 SD |  | 1.09 (0.91,1.31) |  | 1.10 (0.91,1.32) | 1.09 (0.91,1.31) |  | 1.10 (0.91,1.32) |
|  | PDA per *adjusted* 1 SD |  |  | 1.14 (0.94,1.38) |  |  | 1.14 (0.94,1.38) |  |
|  | NDA per *adjusted* 1 SD |  | 0.93 (0.78,1.12) |  |  | 0.93 (0.77,1.12) |  |  |
|  |  |  |  |  |  |  |  |  |
| Interval cases | BIC, AUC | 240, 0.59 | 230, 0.68 | 226, 0.67 | 230, 0.66 | 224, 0.65 | 219, 0.65 | 224, 0.63 |
| (99 cases /324 controls) | BMI per 1 SD |  |  |  |  |  |  |  |
|  | At age 50 years | 0.78 (0.50,1.23) | 0.72 (0.45,1.15) | 0.72 (0.45,1.15) | 0.72 (0.45,1.15) |  |  |  |
|  | At age 70 years | 1.57 (1.05,2.35) | 1.67 (1.07,2.62) | 1.62 (1.05,2.52) | 1.57 (1.03,2.39) |  |  |  |
|  | P for interaction^a^ | 0.04 | 0.02 | 0.03 | 0.03 |  |  |  |
|  | DA per *adjusted* 1 SD |  | 1.66 (1.26,2.19) |  | 1.69 (1.28,2.22) | 1.63 (1.25,2.13) |  | 1.66 (1.28,2.16) |
|  | PDA per *adjusted* 1 SD |  |  | 1.89 (1.39,2.56) |  |  | 1.84 (1.37,2.46) |  |
|  | NDA per *adjusted* 1 SD |  | 0.72 (0.55,0.94) |  |  | 0.74 (0.57,0.96) |  |  |

Abbreviations: AUC, area under the receiver operating characteristic curve; BIC, Bayesian information criterion; BMI, body mass index; CI, confidence interval; DA, dense area; NDA, non-dense area; OPERA, Odds per Adjusted Standard Deviation; PDA, percent dense area; SD, standard deviation.

All of the estimates from conditional logistic regression are adjusted for age at mammogram and the variables included into the model.

^a^ Likelihood ratio test for the interaction with age at diagnosis

Table S2: Risk of breast cancer for BMI and mammographic measures by detection mode and tumor size, excluding HRT users

|  |  | OPERA (95% CI) | | | | | | |
| --- | --- | --- | --- | --- | --- | --- | --- | --- |
|  |  | BMI | BMI+DA+NDA | BMI+PDA | BMI+DA | DA+NDA | PDA | DA |
| Screen detected, small tumours | BIC, AUC | 304, 0.67 | 315, 0.68 | 309, 0.68 | 310, 0.68 | 307, 0.66 | 301, 0.66 | 302, 0.66 |
| (139 cases /387 controls) | BMI per 1 SD |  |  |  |  |  |  |  |
|  | At age 50 years | 0.80 (0.51,1.24) | 0.79 (0.50,1.23) | 0.80 (0.51,1.26) | 0.80 (0.51,1.25) |  |  |  |
|  | At age 70 years | 1.31 (0.99,1.74) | 1.31 (0.99,1.73) | 1.31 (0.99,1.74) | 1.31 (0.99,1.74) |  |  |  |
|  | P for interaction^a^ | 0.07 | 0.07 | 0.08 | 0.08 |  |  |  |
|  | DA per *adjusted* 1 SD |  | 1.03 (0.83,1.27) |  | 1.03 (0.84,1.28) | 1.03 (0.83,1.27) |  | 1.03 (0.84,1.28) |
|  | PDA per *adjusted* 1 SD |  |  | 1.09 (0.88,1.36) |  |  | 1.09 (0.88,1.36) |  |
|  | NDA per *adjusted* 1 SD |  | 0.91 (0.75,1.12) |  |  | 0.91 (0.75,1.12) |  |  |
|  |  |  |  |  |  |  |  |  |
| Screen detected, large tumours | BIC, AUC | 82, 0.62 | 90, 0.67 | 85, 0.65 | 85, 0.67 | 81, 0.67 | 77, 0.66 | 76, 0.67 |
| (31 cases /102 controls) | BMI per 1 SD |  |  |  |  |  |  |  |
|  | At age 50 years | 0.70 (0.25,1.94) | 0.70 (0.24,2.00) | 0.71 (0.25,1.98) | 0.70 (0.24,1.99) |  |  |  |
|  | At age 70 years | 1.09 (0.62,1.92) | 1.14 (0.63,2.08) | 1.11 (0.62,2.00) | 1.14 (0.63,2.05) |  |  |  |
|  | P for interaction^a^ | 0.47 | 0.44 | 0.47 | 0.44 |  |  |  |
|  | DA per *adjusted* 1 SD |  | 1.33 (0.89,1.98) |  | 1.33 (0.89,2.00) | 1.32 (0.89,1.98) |  | 1.33 (0.89,2.00) |
|  | PDA per *adjusted* 1 SD |  |  | 1.33 (0.85,2.06) |  |  | 1.33 (0.86,2.06) |  |
|  | NDA per *adjusted* 1 SD |  | 1.01 (0.64,1.60) |  |  | 0.99 (0.63,1.55) |  |  |
|  |  |  |  |  |  |  |  |  |
| Interval cases, small tumours | BIC, AUC | 131, 0.69 | 132, 0.76 | 126, 0.76 | 129, 0.74 | 127, 0.72 | 121, 0.73 | 124, 0.70 |
| (52 cases /159 controls) | BMI per 1 SD |  |  |  |  |  |  |  |
|  | At age 50 years | 0.52 (0.25,1.08) | 0.47 (0.21,1.03) | 0.47 (0.21,1.03) | 0.47 (0.22,1.04) |  |  |  |
|  | At age 70 years | 1.60 (0.75,3.39) | 2.08 (0.89,4.85) | 2.02 (0.88,4.66) | 1.85 (0.82,4.15) |  |  |  |
|  | P for interaction^a^ | 0.07 | 0.03 | 0.03 | 0.04 |  |  |  |
|  | DA per *adjusted* 1 SD |  | 1.69 (1.14,2.51) |  | 1.69 (1.14,2.52) | 1.59 (1.09,2.32) |  | 1.61 (1.10,2.36) |
|  | PDA per *adjusted* 1 SD |  |  | 1.91 (1.24,2.96) |  |  | 1.77 (1.18,2.67) |  |
|  | NDA per *adjusted* 1 SD |  | 0.76 (0.53,1.10) |  |  | 0.80 (0.57,1.13) |  |  |
|  |  |  |  |  |  |  |  |  |
| Interval cases, large tumours | BIC, AUC | 107, 0.69 | 103, 0.80 | 99, 0.79 | 102, 0.78 | 93, 0.80 | 89, 0.79 | 92, 0.78 |
| (41 cases /151 controls) | BMI per 1 SD |  |  |  |  |  |  |  |
|  | At age 50 years | 1.30 (0.64,2.63) | 1.04 (0.51,2.15) | 1.04 (0.51,2.12) | 1.05 (0.51,2.17) |  |  |  |
|  | At age 70 years | 1.29 (0.73,2.26) | 1.13 (0.56,2.27) | 1.10 (0.56,2.19) | 1.11 (0.60,2.07) |  |  |  |
|  | P for interaction^a^ | 0.98 | 0.89 | 0.91 | 0.92 |  |  |  |
|  | DA per *adjusted* 1 SD |  | 1.91 (1.21,2.99) |  | 1.91 (1.24,2.93) | 1.95 (1.27,3.01) |  | 1.96 (1.30,2.95) |
|  | PDA per *adjusted* 1 SD |  |  | 2.28 (1.35,3.86) |  |  | 2.33 (1.41,3.87) |  |
|  | NDA per *adjusted* 1 SD |  | 0.64 (0.40,1.03) |  |  | 0.65 (0.41,1.03) |  |  |

Abbreviations: AUC, area under the receiver operating characteristic curve; BIC, Bayesian information criterion; BMI, body mass index; CI, confidence interval; DA, dense area; NDA, non-dense area; OPERA, Odds per Adjusted Standard Deviation; PDA, percent dense area; SD, standard deviation.

All of the estimates from conditional logistic regression are adjusted for age at mammogram and the variables included into the model.

^a^ Likelihood ratio test for the interaction with age at diagnosis

Table S3: Risk of interval versus screen-detected cancer for BMI and mammographic measures, excluding HRT users

|  |  | OPERA (95% CI) | | | | | | |
| --- | --- | --- | --- | --- | --- | --- | --- | --- |
|  |  | BMI | BMI+DA+NDA | BMI+PDA | BMI+DA | DA+NDA | PDA | DA |
| All | BIC, AUC | 357, 0.64 | 356, 0.68 | 352, 0.68 | 355, 0.66 | 350, 0.68 | 347, 0.68 | 350, 0.66 |
| 170 SDC/ 99 IC | BMI per 1 SD | 1.04 (0.82,1.30) | 1.01 (0.80,1.28) | 1.01 (0.80,1.28) | 1.01 (0.80,1.28) |  |  |  |
|  | DA per *adjusted* 1 SD |  | 1.36 (1.05,1.76) |  | 1.42 (1.10,1.84) | 1.36 (1.05,1.76) |  | 1.42 (1.10,1.84) |
|  | PDA per *adjusted* 1 SD |  |  | 1.54 (1.18,2.02) |  |  | 1.54 (1.18,2.02) |  |
|  | NDA per *adjusted* 1 SD |  | 0.74 (0.58,0.96) |  |  | 0.75 (0.58,0.96) |  |  |
|  |  |  |  |  |  |  |  |  |
| Small tumors | BIC, AUC | 226, 0.67 | 230, 0.70 | 224, 0.71 | 226, 0.70 | 225, 0.71 | 220, 0.71 | 222, 0.70 |
| 139 SDC/ 52 IC | BMI per 1 SD | 0.88 (0.63,1.22) | 0.91 (0.65,1.27) | 0.90 (0.65,1.25) | 0.88 (0.63,1.22) |  |  |  |
|  | DA per *adjusted* 1 SD |  | 1.41 (0.99,2.00) |  | 1.46 (1.02,2.08) | 1.41 (0.99,2.00) |  | 1.46 (1.02,2.07) |
|  | PDA per *adjusted* 1 SD |  |  | 1.59 (1.10,2.30) |  |  | 1.60 (1.10,2.31) |  |
|  | NDA per *adjusted* 1 SD |  | 0.78 (0.57,1.09) |  |  | 0.77 (0.56,1.07) |  |  |
|  |  |  |  |  |  |  |  |  |
| Large tumors | BIC, AUC | 109, 0.60 | 112, 0.70 | 110, 0.69 | 111, 0.63 | 108, 0.69 | 105, 0.68 | 107, 0.64 |
| 31 SDC/ 41 IC | BMI per 1 SD | 1.09 (0.74,1.62) | 0.91 (0.58,1.42) | 0.97 (0.64,1.49) | 1.03 (0.68,1.54) |  |  |  |
|  | DA per *adjusted* 1 SD |  | 1.35 (0.82,2.22) |  | 1.40 (0.86,2.28) | 1.33 (0.81,2.18) |  | 1.41 (0.88,2.27) |
|  | PDA per *adjusted* 1 SD |  |  | 1.64 (0.96,2.80) |  |  | 1.63 (0.97,2.73) |  |
|  | NDA per *adjusted* 1 SD |  | 0.60 (0.34,1.08) |  |  | 0.62 (0.35,1.10) |  |  |

Abbreviations: AUC, area under the receiver operating characteristic curve; BIC, Bayesian information criterion; BMI, body mass index; CI, confidence interval; DA, dense area; IC, interval cases; NDA, non-dense area; OPERA, Odds per Adjusted Standard Deviation; PDA, percent dense area; SD, standard deviation; SDC, screen-detected cases.

All of the estimates from unconditional logistic regression are adjusted for age at mammogram and the variables included into the model.

Table S4: Risk of breast cancer for BMI and mammographic measures by detection mode, exclude cases (& matced controls) diagnosed within 2 years from mammogram

|  |  | OPERA (95% CI) | | | | | | |
| --- | --- | --- | --- | --- | --- | --- | --- | --- |
|  |  | BMI | BMI+DA+NDA | BMI+PDA | BMI+DA | DA+NDA | PDA | DA |
| Screen detected cases | BIC, AUC | 584, 0.65 | 592, 0.66 | 586, 0.66 | 586, 0.66 | 585, 0.64 | 578, 0.64 | 579, 0.64 |
| (240 cases /614 controls) | BMI per 1 SD |  |  |  |  |  |  |  |
|  | At age 50 years | 0.79 (0.53,1.20) | 0.81 (0.54,1.23) | 0.82 (0.54,1.24) | 0.81 (0.54,1.23) |  |  |  |
|  | At age 70 years | 1.26 (1.04,1.53) | 1.27 (1.05,1.54) | 1.27 (1.04,1.54) | 1.27 (1.05,1.54) |  |  |  |
|  | P for interaction^a^ | 0.06 | 0.07 | 0.08 | 0.07 |  |  |  |
|  | DA per *adjusted* 1 SD |  | 1.18 (1.02,1.37) |  | 1.19 (1.02,1.38) | 1.18 (1.02,1.36) |  | 1.18 (1.02,1.37) |
|  | PDA per *adjusted* 1 SD |  |  | 1.20 (1.03,1.40) |  |  | 1.20 (1.03,1.40) |  |
|  | NDA per *adjusted* 1 SD |  | 0.96 (0.82,1.13) |  |  | 0.96 (0.82,1.13) |  |  |
|  |  |  |  |  |  |  |  |  |
| Interval cases | BIC, AUC | 334, 0.56 | 303, 0.73 | 300, 0.73 | 310, 0.70 | 292, 0.73 | 289, 0.73 | 299, 0.70 |
| (124 cases /368 controls) | BMI per 1 SD |  |  |  |  |  |  |  |
|  | At age 50 years | 0.94 (0.60,1.47) | 0.77 (0.48,1.23) | 0.77 (0.48,1.23) | 0.78 (0.49,1.26) |  |  |  |
|  | At age 70 years | 1.00 (0.73,1.37) | 0.99 (0.71,1.38) | 0.99 (0.71,1.37) | 0.98 (0.71,1.35) |  |  |  |
|  | P for interaction^a^ | 0.85 | 0.44 | 0.46 | 0.51 |  |  |  |
|  | DA per *adjusted* 1 SD |  | 1.87 (1.46,2.40) |  | 1.91 (1.49,2.44) | 1.82 (1.43,2.32) |  | 1.86 (1.46,2.36) |
|  | PDA per *adjusted* 1 SD |  |  | 2.20 (1.67,2.89) |  |  | 2.14 (1.64,2.78) |  |
|  | NDA per *adjusted* 1 SD |  | 0.65 (0.51,0.83) |  |  | 0.66 (0.52,0.84) |  |  |

Abbreviations: AUC, area under the receiver operating characteristic curve; BIC, Bayesian information criterion; BMI, body mass index; CI, confidence interval; DA, dense area; NDA, non-dense area; OPERA, Odds per Adjusted Standard Deviation; PDA, percent dense area; SD, standard deviation.

All of the estimates from conditional logistic regression are adjusted for age at mammogram and the variables included into the model.

^a^ Likelihood ratio test for the interaction with age at diagnosis

Table S5: Risk of breast cancer for BMI and mammographic measures by detection mode and tumor size, exclude cases (& matced controls) diagnosed within 2 years from mammogram

|  |  | OPERA (95% CI) | | | | | | |
| --- | --- | --- | --- | --- | --- | --- | --- | --- |
|  |  | BMI | BMI+DA+NDA | BMI+PDA | BMI+DA | DA+NDA | PDA | DA |
| Screen detected, small tumours | BIC, AUC | 456, 0.67 | 468, 0.67 | 461, 0.67 | 462, 0.67 | 461, 0.64 | 454, 0.64 | 455, 0.64 |
| (188 cases /475 controls) | BMI per 1 SD |  |  |  |  |  |  |  |
|  | At age 50 years | 0.79 (0.50,1.26) | 0.80 (0.50,1.28) | 0.81 (0.51,1.29) | 0.80 (0.50,1.28) |  |  |  |
|  | At age 70 years | 1.29 (1.04,1.60) | 1.30 (1.05,1.60) | 1.29 (1.05,1.60) | 1.30 (1.05,1.60) |  |  |  |
|  | P for interaction^a^ | 0.08 | 0.08 | 0.09 | 0.08 |  |  |  |
|  | DA per *adjusted* 1 SD |  | 1.08 (0.91,1.28) |  | 1.09 (0.92,1.29) | 1.08 (0.91,1.27) |  | 1.08 (0.91,1.28) |
|  | PDA per *adjusted* 1 SD |  |  | 1.10 (0.93,1.31) |  |  | 1.10 (0.93,1.30) |  |
|  | NDA per *adjusted* 1 SD |  | 0.97 (0.82,1.16) |  |  | 0.98 (0.82,1.16) |  |  |
|  |  |  |  |  |  |  |  |  |
| Screen detected, large tumours | BIC, AUC | 138, 0.60 | 140, 0.70 | 136, 0.70 | 136, 0.70 | 130, 0.71 | 126, 0.70 | 126, 0.70 |
| (49 cases /133 controls) | BMI per 1 SD |  |  |  |  |  |  |  |
|  | At age 50 years | 0.68 (0.25,1.86) | 0.80 (0.28,2.22) | 0.78 (0.28,2.13) | 0.73 (0.25,2.11) |  |  |  |
|  | At age 70 years | 1.10 (0.68,1.78) | 1.06 (0.62,1.81) | 1.07 (0.63,1.82) | 1.08 (0.63,1.83) |  |  |  |
|  | P for interaction^a^ | 0.41 | 0.65 | 0.6 | 0.53 |  |  |  |
|  | DA per *adjusted* 1 SD |  | 1.56 (1.10,2.23) |  | 1.56 (1.10,2.23) | 1.58 (1.11,2.25) |  | 1.58 (1.11,2.26) |
|  | PDA per *adjusted* 1 SD |  |  | 1.62 (1.10,2.39) |  |  | 1.65 (1.12,2.42) |  |
|  | NDA per *adjusted* 1 SD |  | 0.83 (0.56,1.22) |  |  | 0.81 (0.55,1.19) |  |  |
|  |  |  |  |  |  |  |  |  |
| Interval cases, small tumours | BIC, AUC | 175, 0.62 | 174, 0.72 | 168, 0.72 | 172, 0.70 | 165, 0.71 | 159, 0.72 | 163, 0.70 |
| (64 cases /179 controls) | BMI per 1 SD |  |  |  |  |  |  |  |
|  | At age 50 years | 0.86 (0.46,1.60) | 0.72 (0.37,1.39) | 0.72 (0.37,1.39) | 0.76 (0.40,1.47) |  |  |  |
|  | At age 70 years | 0.83 (0.51,1.37) | 0.88 (0.53,1.47) | 0.87 (0.53,1.44) | 0.85 (0.52,1.40) |  |  |  |
|  | P for interaction^a^ | 0.94 | 0.67 | 0.7 | 0.82 |  |  |  |
|  | DA per *adjusted* 1 SD |  | 1.59 (1.15,2.19) |  | 1.60 (1.16,2.22) | 1.55 (1.13,2.13) |  | 1.56 (1.14,2.15) |
|  | PDA per *adjusted* 1 SD |  |  | 1.83 (1.28,2.62) |  |  | 1.77 (1.26,2.50) |  |
|  | NDA per *adjusted* 1 SD |  | 0.73 (0.53,1.01) |  |  | 0.74 (0.54,1.02) |  |  |
|  |  |  |  |  |  |  |  |  |
| Interval cases, large tumours | BIC, AUC | 159, 0.61 | 126, 0.86 | 124, 0.85 | 131, 0.82 | 118, 0.85 | 115, 0.84 | 123, 0.81 |
| (55 cases /174 controls) | BMI per 1 SD |  |  |  |  |  |  |  |
|  | At age 50 years | 1.06 (0.52,2.18) | 0.76 (0.34,1.73) | 0.77 (0.35,1.68) | 0.73 (0.33,1.61) |  |  |  |
|  | At age 70 years | 0.99 (0.62,1.58) | 0.75 (0.40,1.40) | 0.76 (0.42,1.40) | 0.78 (0.45,1.34) |  |  |  |
|  | P for interaction^a^ | 0.89 | 0.97 | 0.99 | 0.9 |  |  |  |
|  | DA per *adjusted* 1 SD |  | 3.22 (1.96,5.29) |  | 3.10 (1.96,4.92) | 2.93 (1.83,4.69) |  | 2.81 (1.82,4.33) |
|  | PDA per *adjusted* 1 SD |  |  | 4.05 (2.30,7.13) |  |  | 3.69 (2.16,6.29) |  |
|  | NDA per *adjusted* 1 SD |  | 0.52 (0.33,0.80) |  |  | 0.51 (0.33,0.79) |  |  |

Abbreviations: AUC, area under the receiver operating characteristic curve; BIC, Bayesian information criterion; BMI, body mass index; CI, confidence interval; DA, dense area; NDA, non-dense area; OPERA, Odds per Adjusted Standard Deviation; PDA, percent dense area; SD, standard deviation.

All of the estimates from conditional logistic regression are adjusted for age at mammogram and the variables included into the model.

^a^ Likelihood ratio test for the interaction with age at diagnosis

Table S6: Risk of interval versus screen-detected cancer for BMI and mammographic measures, exclude cases (& matced controls) diagnosed within 2 years from mammogram

|  |  | OPERA (95% CI) | | | | | | |
| --- | --- | --- | --- | --- | --- | --- | --- | --- |
|  |  | BMI | BMI+DA+NDA | BMI+PDA | BMI+DA | DA+NDA | PDA | DA |
| All | BIC, AUC | 475, 0.61 | 459, 0.69 | 457, 0.68 | 466, 0.65 | 455, 0.68 | 453, 0.67 | 462, 0.65 |
| 240 SDC/ 124 IC | BMI per 1 SD | 0.87 (0.70,1.08) | 0.85 (0.68,1.06) | 0.86 (0.69,1.07) | 0.86 (0.69,1.07) |  |  |  |
|  | DA per *adjusted* 1 SD |  | 1.47 (1.17,1.86) |  | 1.55 (1.23,1.95) | 1.47 (1.16,1.85) |  | 1.53 (1.22,1.93) |
|  | PDA per *adjusted* 1 SD |  |  | 1.80 (1.40,2.31) |  |  | 1.79 (1.40,2.29) |  |
|  | NDA per *adjusted* 1 SD |  | 0.65 (0.52,0.82) |  |  | 0.66 (0.52,0.83) |  |  |
|  |  |  |  |  |  |  |  |  |
| Small tumors | BIC, AUC | 290, 0.64 | 290, 0.69 | 285, 0.69 | 290, 0.66 | 288, 0.68 | 283, 0.67 | 288, 0.65 |
| 188 SDC/ 49 IC | BMI per 1 SD | 0.70 (0.51,0.97) | 0.75 (0.55,1.03) | 0.75 (0.54,1.03) | 0.72 (0.52,1.00) |  |  |  |
|  | DA per *adjusted* 1 SD |  | 1.45 (1.05,2.00) |  | 1.50 (1.09,2.07) | 1.48 (1.08,2.03) |  | 1.53 (1.11,2.09) |
|  | PDA per *adjusted* 1 SD |  |  | 1.74 (1.24,2.45) |  |  | 1.80 (1.28,2.52) |  |
|  | NDA per *adjusted* 1 SD |  | 0.71 (0.53,0.95) |  |  | 0.70 (0.52,0.93) |  |  |
|  |  |  |  |  |  |  |  |  |
| Large tumors | BIC, AUC | 156 0.57 | 155, 0.69 | 154, 0.67 | 157, 0.62 | 151, 0.69 | 149, 0.66 | 153, 0.62 |
| 64 SDC/ 55 IC | BMI per 1 SD | 1.04 (0.73,1.48) | 0.89 (0.59,1.33) | 0.93 (0.64,1.37) | 0.97 (0.68,1.41) |  |  |  |
|  | DA per *adjusted* 1 SD |  | 1.41 (0.92,2.16) |  | 1.46 (0.97,2.20) | 1.38 (0.91,2.11) |  | 1.45 (0.97,2.17) |
|  | PDA per *adjusted* 1 SD |  |  | 1.80 (1.14,2.86) |  |  | 1.78 (1.13,2.79) |  |
|  | NDA per *adjusted* 1 SD |  | 0.52 (0.31,0.88) |  |  | 0.53 (0.32,0.89) |  |  |

Abbreviations: AUC, area under the receiver operating characteristic curve; BIC, Bayesian information criterion; BMI, body mass index; CI, confidence interval; DA, dense area; IC, interval cases; NDA, non-dense area; OPERA, Odds per Adjusted Standard Deviation; PDA, percent dense area; SD, standard deviation; SDC, screen-detected cases.

All of the estimates from unconditional logistic regression are adjusted for age at mammogram and the variables included into the model.

Table S7: Risk of breast cancer for BMI and mammographic measures by detection mode, exclude cases diagnosed between one to two years after negative screening, and their matching controls

|  |  | OPERA (95% CI) | | | | | | |
| --- | --- | --- | --- | --- | --- | --- | --- | --- |
|  |  | BMI | BMI+DA+NDA | BMI+PDA | BMI+DA | DA+NDA | PDA | DA |
| Interval cases | BIC, AUC | 189, 0.61 | 176, 0.74 | 172, 0.73 | 180, 0.70 | 165, 0.74 | 162, 0.74 | 170, 0.70 |
| (63 cases /195 controls) | BMI per 1 SD |  |  |  |  |  |  |  |
|  | At age 50 years | 1.38 (0.82,2.32) | 1.02 (0.59,1.76) | 1.02 (0.59,1.77) | 1.11 (0.64,1.91) |  |  |  |
|  | At age 70 years | 0.74 (0.41,1.34) | 0.84 (0.45,1.58) | 0.78 (0.42,1.46) | 0.74 (0.39,1.37) |  |  |  |
|  | P for interaction^a^ | 0.19 | 0.71 | 0.59 | 0.41 |  |  |  |
|  | DA per *adjusted* 1 SD |  | 1.68 (1.22,2.32) |  | 1.78 (1.30,2.44) | 1.66 (1.22,2.27) |  | 1.77 (1.31,2.41) |
|  | PDA per *adjusted* 1 SD |  |  | 2.16 (1.50,3.09) |  |  | 2.14 (1.51,3.02) |  |
|  | NDA per *adjusted* 1 SD |  | 0.58 (0.40,0.82) |  |  | 0.57 (0.40,0.81) |  |  |

Abbreviations: AUC, area under the receiver operating characteristic curve; BIC, Bayesian information criterion; BMI, body mass index; CI, confidence interval; DA, dense area; NDA, non-dense area; OPERA, Odds per Adjusted Standard Deviation; PDA, percent dense area; SD, standard deviation.

All of the estimates from conditional logistic regression are adjusted for age at mammogram and the variables included into the model.

^a^ Likelihood ratio test for the interaction with age at diagnosis

Table S8: Risk of breast cancer for BMI and mammographic measures by detection mode and tumor size, exclude cases diagnosed between one to two years after negative screening, and their matching controls

|  |  | OPERA (95% CI) | | | | | | |
| --- | --- | --- | --- | --- | --- | --- | --- | --- |
|  |  | BMI | BMI+DA+NDA | BMI+PDA | BMI+DA | DA+NDA | PDA | DA |
| Interval cases, small tumours | BIC, AUC | 99, 0.64 | 101, 0.73 | 99, 0.72 | 102, 0.69 | 92, 0.73 | 90, 0.71 | 93, 0.68 |
| (32 cases /95 controls) | BMI per 1 SD |  |  |  |  |  |  |  |
|  | At age 50 years | 0.92 (0.43,1.93) | 0.77 (0.34,1.74) | 0.76 (0.33,1.74) | 0.83 (0.37,1.83) |  |  |  |
|  | At age 70 years | 0.86 (0.39,1.92) | 0.92 (0.39,2.18) | 0.84 (0.37,1.88) | 0.84 (0.38,1.85) |  |  |  |
|  | P for interaction^a^ | 0.93 | 0.8 | 0.89 | 0.99 |  |  |  |
|  | DA per *adjusted* 1 SD |  | 1.26 (0.84,1.90) |  | 1.34 (0.90,2.00) | 1.21 (0.82,1.79) |  | 1.29 (0.88,1.89) |
|  | PDA per *adjusted* 1 SD |  |  | 1.62 (1.06,2.48) |  |  | 1.54 (1.03,2.29) |  |
|  | NDA per *adjusted* 1 SD |  | 0.58 (0.38,0.91) |  |  | 0.59 (0.38,0.91) |  |  |
|  |  |  |  |  |  |  |  |  |
| Interval cases, large tumours | BIC, AUC | 93, 0.69 | 84, 0.84 | 78, 0.84 | 81, 0.83 | 74, 0.84 | 68, 0.84 | 72, 0.83 |
| (30 cases /97 controls) | BMI per 1 SD |  |  |  |  |  |  |  |
|  | At age 50 years | 2.27 (0.91,5.64) | 1.22 (0.51,2.90) | 1.14 (0.47,2.73) | 1.33 (0.54,3.25) |  |  |  |
|  | At age 70 years | 0.55 (0.20,1.51) | 0.84 (0.29,2.45) | 0.88 (0.30,2.59) | 0.69 (0.21,2.27) |  |  |  |
|  | P for interaction^a^ | 0.06 | 0.64 | 0.76 | 0.46 |  |  |  |
|  | DA per *adjusted* 1 SD |  | 2.77 (1.49,5.14) |  | 2.91 (1.58,5.35) | 2.86 (1.57,5.24) |  | 3.07 (1.69,5.55) |
|  | PDA per *adjusted* 1 SD |  |  | 3.98 (1.82,8.69) |  |  | 4.14 (1.96,8.73) |  |
|  | NDA per *adjusted* 1 SD |  | 0.61 (0.33,1.12) |  |  | 0.60 (0.33,1.09) |  |  |

Abbreviations: AUC, area under the receiver operating characteristic curve; BIC, Bayesian information criterion; BMI, body mass index; CI, confidence interval; DA, dense area; NDA, non-dense area; OPERA, Odds per Adjusted Standard Deviation; PDA, percent dense area; SD, standard deviation.

All of the estimates from conditional logistic regression are adjusted for age at mammogram and the variables included into the model.

^a^ Likelihood ratio test for the interaction with age at diagnosis

Table S9: Risk of interval versus screen-detected cancer for BMI and mammographic measures, exclude cases diagnosed between one to two years after negative screening, and their matching controls

|  |  | OPERA (95% CI) | | | | | | |
| --- | --- | --- | --- | --- | --- | --- | --- | --- |
|  |  | BMI | BMI+DA+NDA | BMI+PDA | BMI+DA | DA+NDA | PDA | DA |
| All | BIC, AUC | 315, 0.65 | 306, 0.73 | 304, 0.72 | 311, 0.69 | 304, 0.72 | 301, 0.71 | 308, 0.68 |
| 244 SDC/ 63 IC | BMI per 1 SD | 0.78 (0.57,1.05) | 0.75 (0.55,1.02) | 0.76 (0.56,1.03) | 0.75 (0.55,1.03) |  |  |  |
|  | DA per *adjusted* 1 SD |  | 1.59 (1.17,2.14) |  | 1.63 (1.20,2.20) | 1.55 (1.16,2.08) |  | 1.58 (1.18,2.12) |
|  | PDA per *adjusted* 1 SD |  |  | 1.96 (1.40,2.74) |  |  | 1.91 (1.38,2.64) |  |
|  | NDA per *adjusted* 1 SD |  | 0.61 (0.46,0.83) |  |  | 0.63 (0.47,0.84) |  |  |
|  |  |  |  |  |  |  |  |  |
| Small tumors | BIC, AUC | 191, 0.67 | 193, 0.72 | 191, 0.70 | 194, 0.68 | 189, 0.71 | 187, 0.69 | 191, 0.66 |
| 192 SDC/ 32 IC | BMI per 1 SD | 0.74 (0.48,1.15) | 0.77 (0.50,1.17) | 0.77 (0.50,1.19) | 0.75 (0.49,1.16) |  |  |  |
|  | DA per *adjusted* 1 SD |  | 1.25 (0.83,1.87) |  | 1.28 (0.85,1.91) | 1.27 (0.85,1.89) |  | 1.29 (0.87,1.92) |
|  | PDA per *adjusted* 1 SD |  |  | 1.59 (1.03,2.44) |  |  | 1.62 (1.06,2.47) |  |
|  | NDA per *adjusted* 1 SD |  | 0.62 (0.43,0.89) |  |  | 0.62 (0.44,0.88) |  |  |
|  |  |  |  |  |  |  |  |  |
| Large tumors | BIC, AUC | 116, 0.62 | 114, 0.75 | 111, 0.73 | 114, 0.69 | 111, 0.72 | 108, 0.71 | 111, 0.67 |
| 49 SDC/ 30 IC | BMI per 1 SD | 0.93 (0.59,1.45) | 0.73 (0.44,1.22) | 0.76 (0.45,1.25) | 0.80 (0.48,1.31) |  |  |  |
|  | DA per *adjusted* 1 SD |  | 1.85 (1.06,3.23) |  | 1.86 (1.07,3.23) | 1.69 (1.00,2.85) |  | 1.73 (1.04,2.89) |
|  | PDA per *adjusted* 1 SD |  |  | 2.45 (1.27,4.73) |  |  | 2.19 (1.20,3.99) |  |
|  | NDA per *adjusted* 1 SD |  | 0.48 (0.25,0.94) |  |  | 0.53 (0.28,1.00) |  |  |

Abbreviations: AUC, area under the receiver operating characteristic curve; BIC, Bayesian information criterion; BMI, body mass index; CI, confidence interval; DA, dense area; IC, interval cases; NDA, non-dense area; OPERA, Odds per Adjusted Standard Deviation; PDA, percent dense area; SD, standard deviation; SDC, screen-detected cases.

All of the estimates from unconditional logistic regression are adjusted for age at mammogram and the variables included into the model.
